# “Scientific evidence” can suffer from methodological biases: Outside the scientific scene, criteria need to be well-defined to determine what is scientifically confirmed

**DOI:** 10.1016/j.clinsp.2022.100147

**Published:** 2022-12-16

**Authors:** Newton Kara-Junior

**Affiliations:** Universidade de São Paulo, São Paulo, SP, Brazil

In Medicine, the term “scientific evidence” is no longer a designation used preferentially by doctors and researchers. Regulatory agencies and the judiciary apply it also, in order to support administrative and legal decisions.^1^

We believe, however, that some considerations need to be made about the interpretation of the statement “scientific evidence”, at the risk that generalizing the term would compromise its meaning.

The qualification “scientifically proven” can be subjective and imprecise, when used without extensive critical analysis of the literature, because, at the limit of interpretation, it allows us to show that the conclusions of a single scientific article, chosen without selection criteria, are true. Each clinical research, in general, leads to the publication of a scientific article, which only “adds a brick to the wall of knowledge” on the subject. The “scientific truth” arises from the interpretation of this “wall”, that is, from the critical analysis of all articles published on the subject, which has been resulting from research with methodological accuracy. The type of study that best represents the “scientific truth” is the meta-analysis, which can also have its results contested due to technical limitations.^2^

In the meta-analysis, the evidence available in the literature is synthesized, statistically analyzed, and interpreted to answer a question, considering that there is often research indicating that treatment A is better than treatment B and vice versa.

As there are rules and technical limitations for writing meta-analyses, it is not always possible to use them to answer all clinical questions. On the other hand, the selection, random or not, of a series of articles on a topic, can include research with methodological errors that compromise their results, even after they have gone through peer review and have been published in scientific journals. That selection of a series of articles can also be influenced by confirmation bias, in which the compiler tends to value studies that speak in favor of their belief.^3^

Another important aspect concerns the clinical significance of the research results. It is common for good scientific articles to point out that the effectiveness of one treatment, in relation to another, is statistically significant. However, this does not mean that it is necessarily clinically significant. The clinical relevance of a research result is initially analyzed, in a subjective way, by the author (who may be influenced by interpretation bias) and, later, by the medical community, according to their values and perceptions.^4^ This is also, why an article, which represents only a “brick”, is not enough to support clinical practice.

It is also important to discuss the cost-effectiveness of treatments, as there are therapies with scientific evidence of being slightly more effective than others, at an extremely higher cost, which limits large-scale application. In some cases, there may even be pressure from the pharmaceutical industry to use the most expensive treatment, even if there are no significant clinical advantages.

Thus, in the case of popularization of the terminology “scientific evidence”, it is necessary to establish standards to determine the scientific evidence of new treatments, as well as when establishing who will be responsible for the critical analysis of scientific data. It is better that the evaluators be medical researchers and not judges.

The proposal to value decision-making in public health considering science is promising, especially when there is a rapid advance in the development of new therapies. However, even if the scientific evidence is confirmed, the establishment of treatment does not depend only on the validation of efficacy and safety through clinical trials, it also involves recognition by the medical community and a longer observation of its effects, the so-called proof of time. If only the idea of scientific evidence is accepted, in a generic and unrestricted way, it is likely that disputes of interpretation will occur and that important treatments will be postponed pending judicial decisions. If scientific results cause so much argument and controversy among the scientific community itself, imagine having lawyers added to the quarrel.

Science and its results, in addition to being ephemeral, are too complex to serve as a single, inflexible reference for making administrative decisions. It is important to have a scientific background, but it must be part of a broader evaluation process. Therefore, we consider that, outside the scientific environment, criteria should be defined for the interpretation of what is scientifically proven.

## Conflicts of interest

The authors declare no conflicts of interest.
